# Histopathological and Biochemical Comparative Study of Copper Oxide Nanoparticles and Copper Sulphate Toxicity in Male Albino Mice Reproductive System

**DOI:** 10.1155/2022/4877637

**Published:** 2022-05-16

**Authors:** Manal M. S. AL-Musawi, Hanady Al-Shmgani, Genan A. Al-Bairuty

**Affiliations:** ^1^Biology Department, College of Education for Pure Science/Ibn al-Haitham, University of Baghdad, Baghdad, Iraq; ^2^Department of Microbiology, College of Science, AlKarkh University of Science, Baghdad, Iraq

## Abstract

Copper (Cu) is an essential trace element for the efficient functioning of living organisms. Cu can enter the body in different ways, and when it surpasses the range of biological tolerance, it can have negative consequences. The use of different nanoparticles, especially metal oxide nanoparticles, is increasingly being expanded in the fields of industry and biomedical materials. However, the impact of these nanoparticles on human health is still not completely elucidated. This comparative study was conducted to evaluate the impacts of copper oxide nanoparticles (CuO NPs) and copper sulphate (CuSO_4_ 0.5 (H_2_O)) on infertility and reproductive function in male albino mice BALB/c. Body weight, the weight of male reproductive organs, malondialdehyde (MDA) level, caspase-3 level, and the presence of Ki67 and CD68, as detected using the amino-histochemistry technique, were investigated. Animals were treated with 25 and 35 mg/kg of CuO NPs and CuSO_4_ 0.5 (H_2_O) by oral gavage for 14 days. The control group was given distilled water by oral gavage. Body weight significantly decreased at the end of experiments in both treated groups in a concentration- and time-dependent manner compared with the control group. Weights of testes and epididymis (head and tail), as well as the weight of the seminal vesicle, showed a significant decrease compared with the control. However, the average weights of the seminal vesicle and prostate significantly increased. Caspase-3 and MDA levels increased in the CuO NP and CuSO_4_ 0.5 (H_2_O) groups compared with the control group, and there was a significant difference between the two concentrations used. Immunohistochemical results detected a significant decrease in Ki67 protein in the treatment groups compared with the control. However, increase in CD68 protein was found in groups treated with CuO NPs and CuSO_4_ 0.5 (H_2_O) compared with the control group. Overall, this *in vivo* comparative study of CuO NPs and CuSO_4_ 0.5 (H_2_O) showed that oral intake of copper NPs at 25 and 23 mg/kg was safer to the mice reproductive system than CuSO_4_ 0.5 (H_2_O) at the same dose. CuSO_4_ 0.5 (H_2_O) significantly influenced the histopathological and toxicological alteration responses.

## 1. Introduction

Nanotechnology is a rapidly developing science that has many applications in industry, agriculture, and medicine. Nanoparticles (NPs) are defined as particles 1–100 nm in size that behave as whole units with respect to their transport and properties [1]. The impact of NPs on health and environment is still not completely understood. Copper (Cu) is an essential element required for normal physiological functioning and is used for drug/xenobiotic metabolism, carbohydrate metabolism, and the antioxidant defense system that are necessary for human and animal existence [2, 3]. Cu can enter the body through different ways, i.e., inhalation, consumption of food and water, and dermal contact with air, water, and soil that contains Cu. When intake of Cu exceeds the range of biological tolerance, it can cause adverse effects, including damage to liver, kidney, immune system, and gastrointestinal distress [4, 5].

Copper oxide nanoparticles (CuO NPs) are well known and widely used as metal oxides in many diverse products. They are also used as catalysts in electronics and cosmetic products. The increased use of CuO NPs and the long-term damage they potentially cause to humans have prompted researchers to increase their efforts to determine CuO NPs' adverse effects *in vitro* and *in vivo* [6]. Increasing evidence has shown that the toxicity of CuNPs is mediated by the generation of oxidative stress that involves the induction of lipid peroxidation and ROS-dependent DNA damage [7]. The other form of Cu used in this study is the ionic form of copper sulphate, i.e., CuSO_4_ 0.5 (H_2_O), which is an inorganic compound that acts as a potent oxidant and has a long history of use in agriculture, particularly as a fungicide, bactericide, and pesticide against smaller animals like snails [8]. The influence of metal toxicity depends on the physical nature, dose, and period of exposure. The effect may reach the embryos or affect their growth rate. A study [9] investigated the effect of CuO NPs on the embryos of fish after 4 h of exposure and at concentrations of 0.5, 1, and 1.5 mg/L to determine if these increased mortality, delayed hatching, decreased heart rate, and caused a number of deformities in the head, tail, and spine.

Although the CuNPs were synthesized using two distinct types of synthesis forms, and their cytotoxicity effects were compared in [10]. However, copper NPs may or may not be more hazardous than regular forms of copper, and the relative pathological impact of regular copper metal salts and nano forms is still not completely understood. Hence, the present study was conducted to evaluate the effects of two types of copper, metallic copper, and metallic nano-oxides, on the spermatogenesis process, antioxidants, and cell death proteins in the male reproductive system of mice.

## 2. Materials and Methods

### 2.1. Chemicals and Materials

CuO NPs with a particle size of 40 nm and a 99.9% trace metal basis and copper sulphate pentahydrate CuSO_4_ 0.5 (H_2_O) were purchased from Sky Spring Nanomaterial's USA Company. Ki67 and CD68 antibodies were obtained from Biocare Medical, USA. All other reagents were purchased from SkySpring Nanomaterials, USA. Mice caspase-3 kit was obtained from MyBioSource Inc., San Diego, CA, USA (ref: MBS733100).

### 2.2. Experimental Design

Thirty healthy adult male albino mice (*Mus musculus*) weighing 25–30 g were used in this study. Mice were obtained from the National Center for Control and Pharmaceutical Research in Baghdad, Iraq. They were housed in the animal house of the biological department, College of Education for Pure Sciences, Ibn Al-Haitham. Animals were fed with standard commercial pellet and tap water and kept in a standard atmosphere condition. Mice were randomly divided into five groups (*N* = 6 in each). The following were orally administered to the groups of mice for 14 days: group I (GI) served as control group and received distilled water; group II (GII) received 25 mg/kg CuSO_4_ 0.5 (H_2_O); group III (GIII) orally received 25 mg/kg CuO NPs; group IV (G IV) received 35 mg/kg CuSO_4_ 0.5 (H_2_O); and group V (G V) received 35 mg/kg CuO NPs. The two doses were compared using data from a previous study that estimated LD50 in rat (data not shown). The current study was approved by the University of Baghdad Ethics Committee of the College of Education for Pure Sciences (Ibn Al-Haitham), Iraq (approval no. 3 in 2/10/2019).

### 2.3. Characterization of CuO NPs

CuO NP solution was prepared and characterized as described by [11]. In brief, the stock solution was prepared by dissolving CuO NPs in distilled water; after rapid stirring, sonication was done for 5 min. The particle morphology and sizes were determined by transmission electron microscopy (TEM) and scanning electron microscopy (SEM) (JEOL 5600, MA, USA). X-ray diffraction spectroscopy (XRD) (Shimadzu, Japan) was used to demonstrate the surface of the synthesized nanoparticles. UV-visible spectroscopy (UV-Vis) analysis was carried out by UV-visible spectroscopy (U-2910 Hitachi, Tokyo, Japan) and was used along with Fourier transform infrared (FTIR) analysis for characterization (Thermo Fisher Scientific, Madison, USA).

### 2.4. Tissue Preparation and Organ Index Determination

At the end of the experiment, mice were weighed and sacrificed under anesthesia. Testes were dissected and weighed to calculate organ indexes; then, they were either fixed in 10% formalin for histology preparation or homogenized for biochemical experiments [12]. The testicular tissue samples were homogenized in ice-cold sterile phosphate-buffered saline and then centrifuged for 10 min at 4°C at 13,000 rpm. The supernatants were used for biochemical experiments. Organ index was calculated as follows:

(1) $$\begin{array}{c} \frac{\text{organ indexes }\left(\text{\%}\right)=\text{weight of the organ}\left(\text{mg}\right)}{\text{weight of the animal body}\left(\text{g}\right)}\times 100\text{.} \end{array}$$

### 2.5. Histology Study

Fixed testes in 10% formalin were prepared according to the method used in a previous study [13]. Samples were dehydrated through ethanol at a series of ascending concentrations and embedded in paraffin wax after clarifying in xylene. Sections (5 *μ*m slices) were stained with hematoxylin and eosin (H&E). Slides were then examined and photographed by using the light microscope [13] to study the histological structure.

### 2.6. Immunohistochemistry for the Detection of Ki67 and CD68 in Testes Tissue

Samples embedded in paraffin (the section histology study mentioned above) were sections of 5 *μ*m thickness and loaded onto charged slides. After dewaxing and dehydrating, the slides were immersed in antigen retrieval solution and blocked by adding a solution bovine serum albumin (BSA 5%). Sections were incubated with primary antibody of Ki67 or Cd68 (1 : 100 dilutions) overnight at 4°C. Sections were then incubated with secondary antibodies at room temperature for 2 h. Substrate chromogen solution was added, and sections were stained with hematoxylin. The expression of proteins of interest was calculated as the percentage of positive cells per total 100 cells in 10 slide fields [14].

### 2.7. Determination of Lipid Peroxidation

Lipid peroxidation was determined according to [15]. Malondialdehyde (MDA) level in the testicular homogenate tissue was measured as follows: 1 ml supernatant of testes homogenized tissue was mixed with 1 ml of trichloroacetic acid 17.5% and 1 ml of 0.6% thiobarbituric acid. The mixture was incubated in a water bath at 100°C for 15 min. After cooling, 1 ml of 70% TCA was added and left to stand at room temperature for 20 min. The sample mixture was then centrifuged at 2000 rpm for 15 min, and the absorbance was measured at 532 nm.

### 2.8. Caspases-3 Activity in Testicular Tissue

ELISA kit was used to determine caspase-3 activity in testicular homogenate according to the manufacturer's instructions.

### 2.9. Fertility Study

In this experiment, 30 healthy adult females that were isolated after smear vaginal swab test were examined every morning to determine the estrus cycle. This was moistened with distilled water after sterilization and was placed in the vaginal opening of the mouse. It was inserted deep into the vaginal opening and moved in a rotational manner so that the cells adhered. Then, the cells were placed on a clean glass slide, brushed, and dyed with methylene blue 0.5% for 2 min. They were washed with water, and a slide cover was placed on them.

Each male was placed with two healthy females in one cage, and they were allowed to mate. Mating was confirmed by examining the vaginal plug and with the presence of sperm in the vaginal swab. Pregnancy was confirmed by recording the weights of females daily, and after the end of the pregnancy period (21 days), the number of births and progeny weights, lengths, and deformities were recorded. Embryos resulting from treated males were evaluated.

### 2.10. Statistical Analysis

For statistical analysis, SPSS (version 24) was used. The data were presented in the form of mean ± standard error (SE). Analysis of variance (one-way ANOVA) was used and the difference between groups indicated using the least significant difference. The significant was accepted at *P* ≤ 0.05.

## 3. Results and Discussion

### 3.1. Characterization of CuO NPs

**Figure 1:**
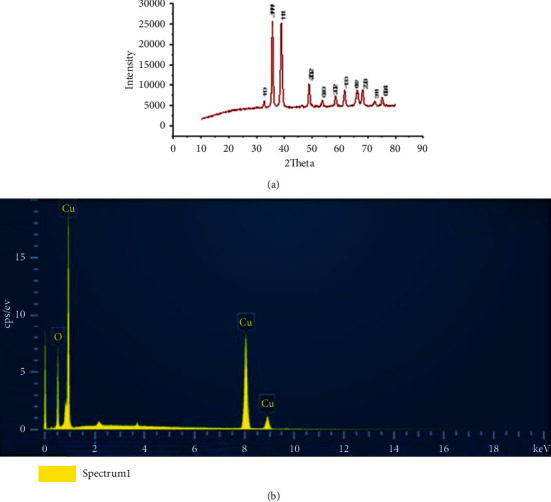
X-ray diffraction (XRD) (a) and energy dispersive X-ray spectroscopy (EDX) (b) of CuO NPs with the presence of the elemental compositions of copper and oxygen.

The crystalline structures of CuO NPs were examined by XRD. Various peaks were shown, indicating the monoclinic crystal. The size of crystallite was calculated using Debye–Scherrer's equation. The reflection peaks observed at 2*θ* = 32.72°, 35.76°, 38.87°, 49.11°, 53.74°, 58.38°, 61.72°, 66.51°, and 68.25° were related to (110), (−111), (111), (−202), (020), (202), (−113), (−311), and (220) plane orientations of CuO, respectively, which was in agreement with the Cu_2_O powder peaks obtained from the International Center of Diffraction Data card (JCPDS file no. 05–0661). The purities of Cu NP powder were determined as 99% using EDX (Figure 1).

**Figure 2:**
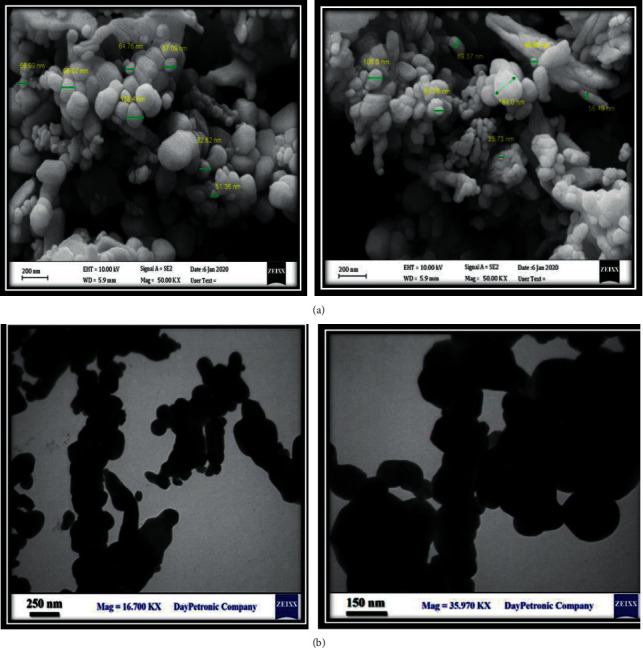
Scanning electron microscopy (SEM) (a) and transmission electron microscopy (TEM) (b) of CuO NPs.

**Figure 3:**
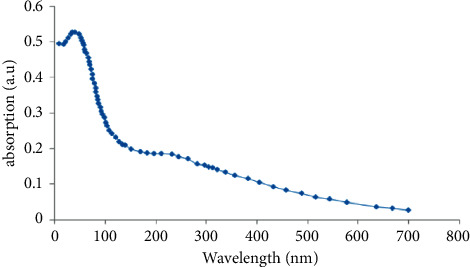
UV-Vis spectra of CuO NPs.

**Figure 4:**
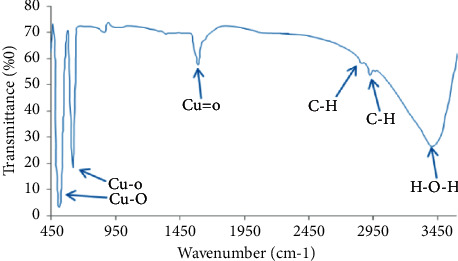
The FTIR spectrum of CuO NPs.

The size and morphology were determined by TEM and SEM. Results showed spherical morphology, and their diameter range was 22 ± 10 nm (Figure 2). Absorption spectra of CuO NPs are shown in Figure 3. The figure shows a strong fundamental absorption edge of approximately 219 nm due to direct transition of electrons, which were acceptable to the formation of CuO NPs. Optical absorption showed that the direct band gap compared with indirect band gap allowed the determination of the crystallinity of a material. Functional groups were determined using FTIR analysis (Figure 4).

The obtained spectra at 640 cm^−1^ were attributed to the characteristic band of monoclinic phase of pure CuO. The peaks at around 3450 cm^−1^ corresponded to O-H stretching vibrations and water. The peaks at 2950–2800 cm^−1^ corresponded to C-H stretching vibration. The bands obtained at 1585 and 1644 cm^−1^ showed the carbonyl C=O stretching bonds.

### 3.2. Body Weight and Organ Index

**Figure 5:**
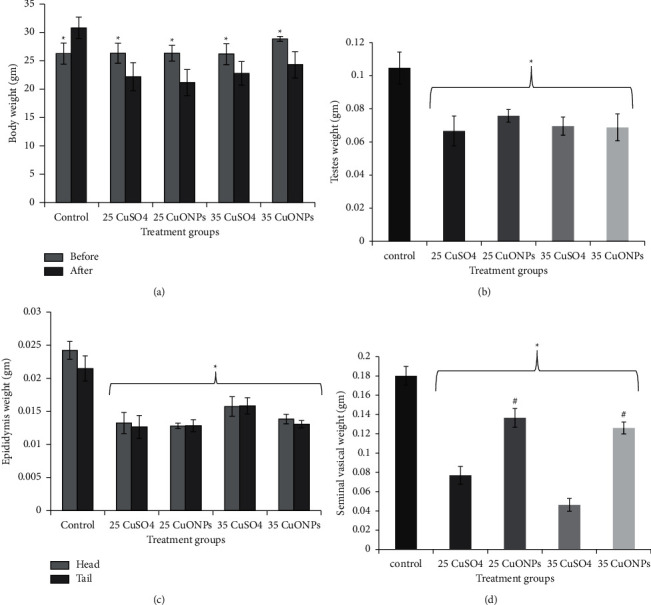
Effect of CuO NPs and CuSO_4_ 0.5 (H_2_O) on rat at different concentrations (25 and 35 mg/kg) after 14 days of exposure on (a) body weight, (b) testes weight, (c) epididymis weight, and (d) seminal vesical weight.

The results showed a significant difference (*P* ≤ 0.05) in body weight before and at the end of experiment. A significant difference was found in the weights of the testes and epididymis (head and tail) in all treatment groups compared with the control group. Seminal vesicles weight results exhibited significant difference between treatment and control groups. Interestingly, a significant difference was found between CuO NPs and CuSO_4_ groups at the same concentration. In contrast, the changes were not significant between the different concentrations for the same group, as shown in Figure 5 and Table 1. Features of toxicity, including weight loss in body and association organs, induced by Cu have been documented by several studies [16]. Male mice were exposed to 0, 100, and 200 mg ZnONP/kg bw/day (particle size of 50 nm) for 7 or 14 days. In all exposed groups, significantly reduced testes, epididymal, seminal vesicle, and prostate weights were observed. The study also showed a decrease in the weight of the reproductive organs of mice treated with CuO NPs [17]. The study [18] showed that copper sulphate at a concentration of 200 mg/kg affected the final weight of rats treated for 30 days. A decrease in the weights of the organs studied (testes, culverts, seminal vesicles, and vas deferens) was observed. Some of the suggested reasons for the decrease in weight were necrosis and degeneration in the reproductive system's cells and tissues. If the weight of the testes depends on the weights of the mass of germ cells, then any decrease in the weight of the testes is caused by the death of germ cells and a defect in the spermatogenesis process [19]. This finding is consistent with our histology results (data not shown). Another suggestion is that the changes and the decrease in weight were due to the accumulation of metals in the testes and associated organs. Thus, toxicity occurs by changing the shape of the germ cells and by affecting spermatogenesis [20–22].

### 3.3. Lipid Peroxidation in Testes

**Figure 6:**
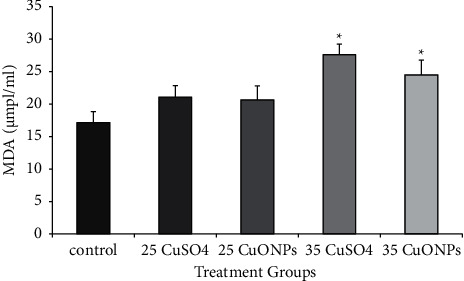
Lipid peroxidation (malondialdehyde) in rat testes homogenate was affected by CuO NPs and CuSO_4_ 0.5 (H_2_O). Data represent the mean ± standard error (SE). ^*∗*^Significantly different (*P* ≤ 0.05) from the control group.

The results of MDA evaluation indicated a significant increase (*P* ≤ 0.05) in CuO NPs and CuSO_4_ at 35 mg/kg groups (25.64 ± 1.31 and 26.49 ± 0.917 *μ*mol/ml, respectively) compared with the control group (17.16 ± 0.97 *μ*mol/ml). The increase in CuSO_4_ at 35 mg/kg was higher than that of CuO NPs at the same concentration, although the difference was not significant (Figure 6). MDA production is a good biomarker of oxidative stress caused by the excessive generation of reactive oxygen species (ROS) [23].

Several studies showed that lipid peroxidation in tissue occurred due to oxidative stress and damage after exposure to metal oxide nanoparticles [24, 25]. The intraperitoneal injection of silver nanoparticles in treated groups caused a significant increase in lipid peroxidation (MDA) and a significant decrease in the activity of antioxidant enzymes, such as GSH and catalase, compared with the control group. This finding indicated the occurrence of oxidative stress in the testicular tissue. The results of [26] showed that the results obtained after exposing pigs to 125 and 250 mg/kg of copper in the form of copper sulphate for 80 days were consistent with the results in the present study, i.e., the levels of MDA increased, and apoptosis and autophagy were promoted in the testes.

A previous study reported a significant increase in MDA in the liver and kidney of rats injected with CuO NPs as a consequence of oxidative stress and lipid peroxidation due to excessive nanocopper accumulation in hepatocytes or nephrocytes; these resulted in mitochondria failure and cell death. Moreover, Arefian et al. [27] found that when rats were exposed to a high dosage of nanoparticles, a significant increase in MDA concentration level occurred. Rats exposed to CuSO_4_ 0.5 (H_2_O) showed an increase in the level of MDA and a decrease in the total antioxidant potential in the liver, renal, and brain tissues [28].

The results of this study showed more effect of copper salt more than NPs at the same concentration, and this may be due to the limiting of agglomeration of NPs decreases the potential increase of mechanical characteristics in NPs. Furthermore, nanoparticle agglomeration has been taken as a broad word that alters the properties of nanocomposites [29]. Also, this may be due to the oxidative state as CuSO_4_ 0.5 (H_2_O) is a strong oxidizing agent, and then it has many negative effects.

### 3.4. Caspase-3 Level

**Figure 7:**
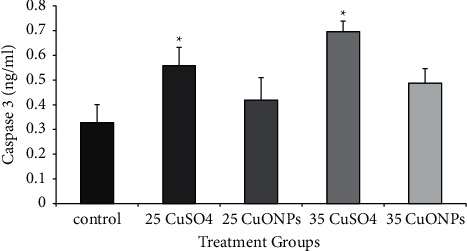
Caspase-3 level in rat testes homogenate affected by CuO NPs and CuSO_4_ 0.5 (H_2_O). Data represent the mean ± standard error (SE). ^*∗*^Significantly different (*P* ≤ 0.05) from the control group.

The results of caspase-3 level in testes homogenate revealed a significant increase in all treated groups compared with the control group. The animals treated with CuSO_4_ at a concentration of 35 mg/kg showed a significant difference in the level of caspase-3 compared with the animals treated with the same concentration, as shown in Figure 7.

Findings of this study confirmed previous studies' findings, which showed that exposure to NPs caused toxic effects and the generation of infection in testes tissue. These, in part, are due to the accumulation of these particles and by the conversion of CuNPs by hydrogen into cupric ions, which are very toxic to tissues [30–32].

A previous study [33] reported the presence of small amounts of these substances in the interstitial tissue of the testes and the adverse effects in the germ. Moreover, the high quantity of unsaturated fatty acids in the germ cell membranes made the germ cell more vulnerable to oxidative stress caused by the presence of an excessive amount of copper [34].

Previous research has shown that NPs can cause cytotoxicity by increasing inflammation, oxidative stress, and apoptosis, as well as by causing damage at the molecular and genetic levels [35].

Necrosis and/or apoptosis associated with metal and nanoparticle accumulation have a deleterious effect on DNA and trigger cell death. A previous study [36] showed that oral demonstration for CuSO_4_ 0.5 (H_2_O) at 50, 100, and 200 mg/kg can cause renal dysfunction and tubular necrosis in the kidney tissues of mice treated with CuSO_4_ 0.5 (H_2_O). The expressions of caspase-9 and caspase-3 were upregulated.

### 3.5. Immunohistochemical Results

**Figure 8:**
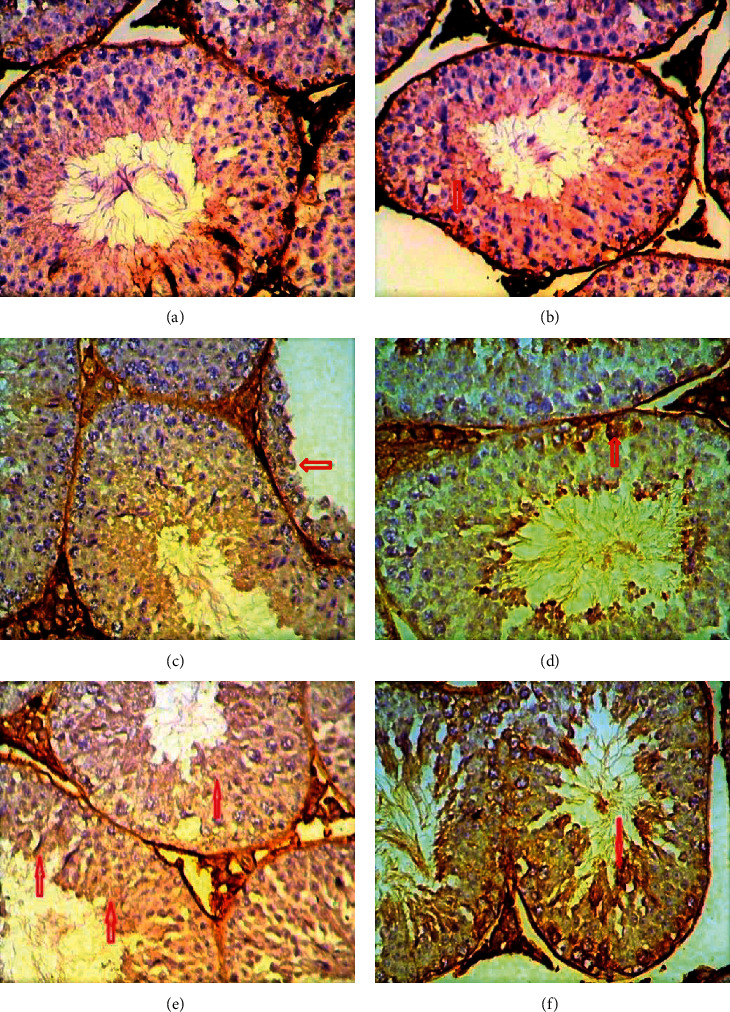
Immunohistochemical staining method detection of Ki67 using a section of mice testis tissue treated with CuSO_4_ and CuO NPs at 25 and 35 mg/kg; weak positive expression: (a, b, d) CuO.T1; (e) CuSO_4_.T2; (f) CuO.T2; (c) CuSO_4_.T1 (x40).

The detection and distribution of Ki67 immune reaction results are elucidated in Table 2 and Figure 8. Spermatogonia and Sertoli cells in the control group testes showed a strong positive reaction, although other spermatocyte stages and Leydig cells revealed a negative reaction. In contrast, weakly or slightly positive response for Ki67 was detected in the spermatogonia and Sertoli cells of testis tissue treated with copper sulphate at a concentration of 35 and 25 mg/kg. Moderate positive reaction was observed in the testes treated with the two concentrations of CuO NPs.

**Figure 9:**
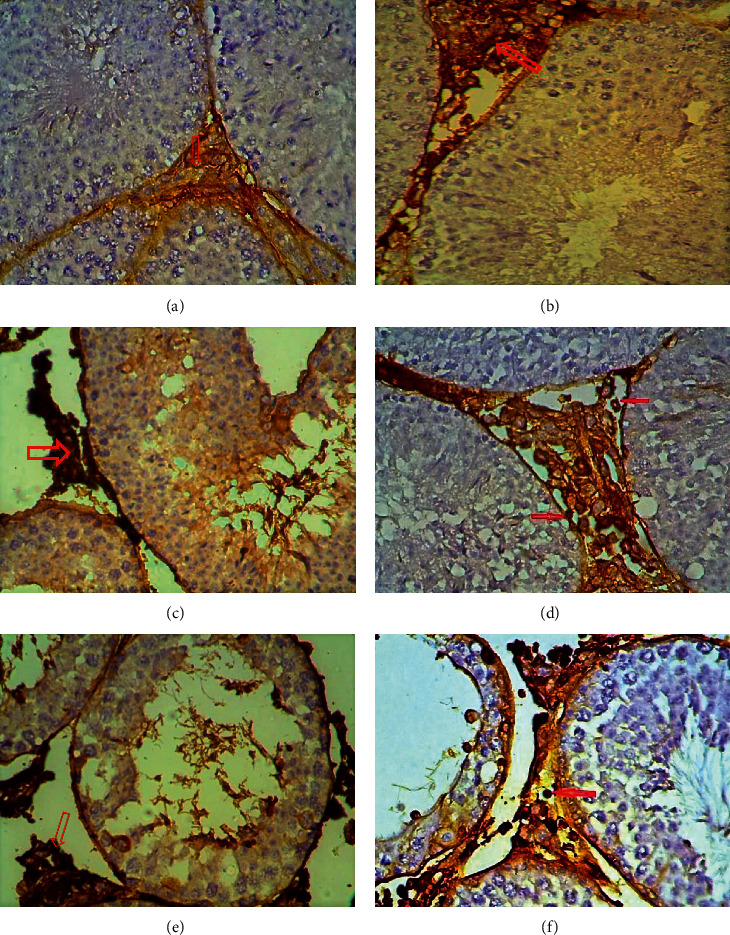
Immunohistochemical staining method detection of CD68 using a section of mice testis tissue subjected to different treatments (CuSO_4_ and CuO NPs at 25 and 35 mg/kg). Strong positive expression: (a) CuSO_4_.T1; (b, c) CuO.T1; (d) CuSO_4_.T2; (e, f) CuO.T2.

Examination of CD68 reaction in testes tissue sections showed negative or very weak positive reaction in the control group, as shown in Figure 9 and Table 2. In contrast, the group treated with CuSO_4_ showed a strong positive expression of CD68 in the Leydig cells, especially at a concentration of 35 mg/kg. However, the reaction expression in Leydig cells and spermatozoa was moderate in testes sections treated with CuO NPs.

The highly positive Ki67 expression in spermatogonia and primary spermatocyte especially type I, as shown in the control group, can be explained by the normal proliferative physiological role of these cells. The decrease in the Ki67 expression in the treated groups was more pronounced in the groups receiving a higher dose. This result may be due to the severe damages caused by copper to the two states used in the present study (cells undergoing spermatogenesis and Sertoli cells). Such damage was the result of the increased production of free radicals. Oxidative stress is one of the important mechanisms underlying NP cytotoxicity [37], and the increase in MDA levels obtained in this study supported this suggestion. These results are different from those obtained by other researchers [38], who reported that overproduction of reactive oxygen species and oxidizing enzyme activation caused damage to cell membrane and mitochondria along with the cytoskeleton of germ cells and triggered apoptosis. Moreover, NPs penetrate through the blood-testis barrier (BTB) formed by tight junctions between Sertoli cells [39]. This result is in contrast with that obtained by a previous study [40]. Immunohistochemical analysis revealed the reduced proliferative activity and differentiation potential of epithelial cells, which was confirmed by the changes in Ki67 expression.

Immunohistochemical detection of testicular macrophages by CD68 antibodies revealed a significant increase in expression in the treated groups. This is a result of the incidence of necrosis and cell death, as shown in the results of this study, i.e., the high level of caspase-3 in the treated groups. This finding supports the results of this study. Macrophages play an important role in immune cells in the testis and play a nutritional role in the testes beyond its normal functions [41]. Moreover, macrophages play a critical role in Leydig cell regeneration [42]. This finding is consistent with an unpublished study of [43], which showed a significant reduction in the number of Leydig cells in the experimental groups, and this was especially prominent at higher concentrations (100 and 200 mg/kg) of silver NPs. The results of this study agreed with the findings of Ahmed et al. [14] who showed that injecting rats with silver nanoparticles at concentrations of 100 and 1000 mg/kg for periods of 7 and 21 days led to an increase in the level of CD68 in the treated groups. These showed the effects of CuO NPs and aqueous CuSO_4_ 0.5 (H_2_O) on the fertility of mice.

### 3.6. Determination of Teratogenicity

**Figure 10:**
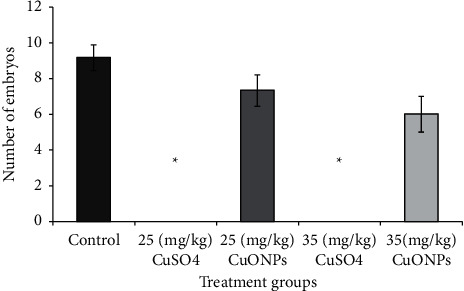
The effect of CuO NPs and CuSO_4_ 0.5 (H_2_O) on fertility and number of fetuses in mice.

The results of the study showed a decrease in the number of embryos of male mice dosed with CuO at concentrations of 25 and 35 mg/kg for 14 days compared with the control group and the incidence of infertility cases for males dosed with CuSO_4_ 0.5 (H_2_O) at both concentrations, as shown in Figure 10. This finding was due to the low number of sperms, thus reducing the chances of fertilizing eggs during mating. This finding is consistent with the results of Ki67, which is an indicator of cell proliferation.

The results of the study showed some abnormalities in the external appearance of newborn mice at the age of 1 day resulting from the mating of males treated with 25 and 35 mg/kg of the CuO NPs for 14 days with healthy females in comparison with the shape of normal fetuses of the control group. The occurrence of abnormalities increased with increasing concentration.

Moreover, the mating of males treated with CuSO_4_ 0.5 (H_2_O) with healthy females did not lead to any births, which proved that sulphate led to infertility, as shown in Figure 10.

**Figure 11:**
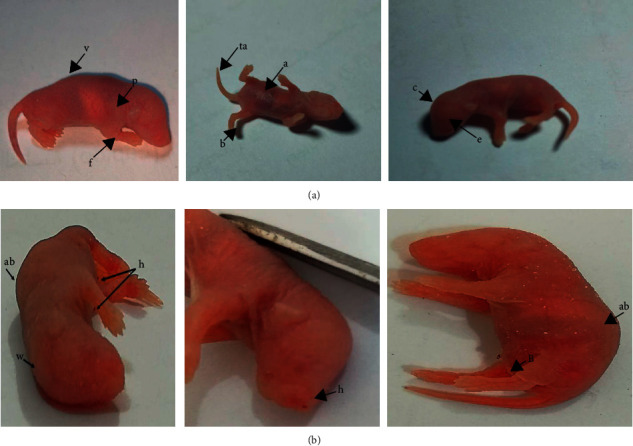
(a) The outward appearance of a normal newborn rat and (b) malformations in newborn mice resulting from the mating of male mice dosed with CuO NP at concentrations of 25 and 35 mg/kg with healthy females. The deformities were represented by the appearance of (h) blood clotting (b) thickening of the skin and deformation of the hind limb, and (a, b) convexity of the column spine.

The shape of normal embryos is represented by a head with a normal cranium, a pinna, eyes, and a trunk that shows the normal shape of the spine from the dorsal side, the tail, the front and rear sides of the fetus, and the anogenital region, as shown in Figure 11.

The abnormalities in mice treated with CuO NP for a period of 14 days were represented by the thinning of the skin, as well as the shrinkage and shortening of the extremities with the occurrence of blood congestion in some fetuses and in different parts of the body, flexion of the hind limb, and curvature of the spine (Figure 11). These are might due to the toxicity of the NPs and its effect on the vitality, concentration, and distortion of sperm in males. From this, it can be suggested that eggs can be fertilized with distorted sperm, leading to the formation of embryos with some abnormalities that may be in physical or sexual characteristics. It has been found that NPs such as gold NPs and TiO2 NPs are extremely toxic to Leydig cells, which is reflected in the levels of sex concentration hormones, and the buildup of nanoparticles leads to a deficit in fertility as well as an effect on offspring's growth [17, 44]; furthermore, alteration in sperm cell motility are also thought to be a cause of embryo teratogens [45], This finding was consistent with that of [46], who showed that when mice were dosed with nanographene and three concentrations of 10, 20, and 30 mg/kg for 14 days, some deformities were observed (e.g., shortening of the limbs, thickening of the skin, and blood clotting in some organs).

CuO NPs can pass through the blood-testis and epithelial barriers that protect reproductive tissues and accumulate in reproductive organs. NP accumulation damages organs (testis, epididymis, and seminal vesicle) by destroying Sertoli, Leydig, and germ cells, resulting in reproductive organ dysfunction that affects sperm quality, quantity, morphology, and motility.

## 4. Conclusion

The toxicity of CuSO_4_ 0.5 (H_2_O) and CuONPs, as well as some causes behind it, has been investigated in the current in vivo study in male reproductive system of mice. It was demonstrated that the pathologies caused by CuSO_4_ 0.5 (H_2_O) were more than copper oxide NPs with the same concentration. The findings also revealed that the impacts caused by copper NPs are significant, necessitating a reduction in the safe level dose for organisms to a nonsignificant level. However, further investigations are needed to distinguish the underling mechanisms associated with direct impact by particle accumulation or in direct influence through secondary oxidative stress during exposure to copper and its NPs.

## Figures and Tables

**Table 1:** Changes in male mice body weight and reproductive organs induced by treatment with copper nanoparticles and copper sulphate.

Treatment groups	Body weight before treatment (gm)	Body weight after treatment (gm)	Testes index (%)	Epididymis index (head) (%)	Epididymis index (tail) (%)	Seminal vesicle index (%)
Control	26.25 ± 1.85	30.82 ± 1.88	0.33 ± 0.13	0.078 ± 0.010	0.069 ± 0.019	0.58 ± 0.18
25 CuSO_4_	26.33 ± 1.75	22.16 ± 2.48 a	0.29 ± 0.07 a	0.059 ± 0.014 a	0.056 ± 0.017 a	0.34 ± 0.10 a
25 CuO NPs	26.33 ± 1.36	21.15 ± 2.29 a	0.36 ± 0.06	0.061 ± 0.010 a	0.060 ± 0.009	0.64 ± 0.08 a
35 CuSO_4_	26.16 ± 1.83	22.78 ± 2.10 a	0.30 ± 0.06	0.069 ± 0.018 a	0.07 ± 0.015	0.20 ± 0.08 a
35 CuO NPs	28.86 ± 0.43	24.28 ± 2.31 a	0.27 ± 0.13 a	0.057 ± 0.006 a	0.054 ± 0.007 a	0.51 ± 0.05

Data represent the mean ± standard deviation (SD). A: significant difference at *P* ≤ 0.05 compared with the control.

**Table 2:** The percentage of immunoreactivity of Ki67 and CD68 proteins in male mice testes induced by treatment with copper nanoparticles and copper sulphate.

Parameter groups	Ki67 immunoreaction (mean ± SD)	CD68 immunoreaction (mean ± SD)
Control	45.00 ± 7.64^a^	10.00 ± 2.89^a^
25 mg/kg CuSO_4_	11.67 ± 1.67^b^	31.67 ± 1.67^b^
25 mg/kg CuO NPs	31.67 ± 1.67^c^	13.33 ± 1.67^c^
35 mg/kg CuSO_4_	8.33 ± 1.67^d^	36.67 ± 4.41^d^
35 mg/kg CuO NPs	35.00 ± 2.87^e^	15.00 ± 5.00^e^

Data represent the mean ± standard deviation (SD). Different letters represent a significant difference at *P* ≤ 0.05.

## Data Availability

The research data used to support the finding of this study are involved within the article.
